# A porcine reproductive and respiratory syndrome virus (PRRSV) vaccine candidate based on the fusion protein of PRRSV glycoprotein 5 and the Toll-like Receptor-5 agonist *Salmonella* Typhimurium FljB

**DOI:** 10.1186/s12917-015-0439-0

**Published:** 2015-05-23

**Authors:** Dan Xiong, Li Song, Xianyue Zhai, Shizhong Geng, Zhiming Pan, Xinan Jiao

**Affiliations:** Jiangsu Key Laboratory of Zoonosis, Yangzhou University, 48 East Wenhui Road, Yangzhou, Jiangsu 225009 China; Jiangsu Co-innovation Center for Prevention and Control of Important Animal Infectious Diseases and Zoonoses, Yangzhou, Jiangsu 225009 China

**Keywords:** PRRSV, Glycoprotein 5, Flagellin, Fusion protein, Vaccine

## Abstract

**Background:**

Porcine reproductive and respiratory syndrome (PRRS) is characterized by severe reproductive failure and severe pneumonia in neonatal pigs and is caused by PRRS virus (PRRSV). Glycoprotein 5 (GP5) from PRRSV is a key inducer of neutralizing antibodies. Flagellin, a toll-like receptor 5 (TLR-5) agonist, is an effective inducer of innate immune responses. This study presents a novel PRRSV vaccine candidate based on the adjuvant effect of *Salmonella* Typhimurium FljB fused with PRRSV GP5.

**Results:**

A truncated rGP5 gene lacking the signal peptide and transmembrane sequences was amplified and inserted into prokaryotic expression vectors, pColdI or pGEX-6p-1. *Salmonella* Typhimurium flagellin *fljB* was amplified and inserted into the plasmid pCold-rGP5, generating recombinant plasmid pCold-rGP5-fljB. Histidine (His)-tagged rGP5 and fusion protein rGP5-FljB were induced with isopropyl-β-d-thiogalactoside, verified by SDS-PAGE and western blotting, and purified via Ni-NTA affinity columns. The TLR-5-specific bioactivity of fusion protein rGP5-FljB was determined by detecting the expression levels of the cytokine IL-8 in HEK293-mTLR5 cells by sandwich ELISA. The purified endotoxin-free proteins were administered intraperitoneally in a C3H/HeJ mouse model. The results show that immunization with the fusion protein rGP5-FljB induced a significantly enhanced GP5-specific and PRRSV-specific IgG response that persisted for almost 5 weeks. Co-administration of the rGP5 with R848 or Alum also yielded a higher IgG response than administration of rGP5 alone. The IgG1/IgG2a ratio in the rGP5-FljB immunization group was significantly higher (9-fold) than that in the rGP5 alone group and was equivalent to the response in the rGP5 + Alum immunization group, suggesting a strong Th2 immune response was induced by the fusion protein.

**Conclusions:**

Purified fusion protein rGP5-FljB is capable of activating the innate immune response, as demonstrated by the results of our TLR-5-specific bioactivity assay, and FljB has adjuvant activity, as shown by the results from our administration of rGP5-FljB in a mouse model. Our findings confirm that FljB could serve as an excellent adjuvant for the production of GP5-specific and PRRSV-specific IgG antibodies as part of an induction of a robust humoral immune response.

## Background

Porcine reproductive and respiratory syndrome (PRRS) has been recognized as one of the most serious infectious diseases of swine since its first appearance in North America in 1987 [1]. The disease is characterized by severe reproductive failure and severe pneumonia in neonatal pigs and is caused by PRRS virus (PRRSV), a member of the *Arteriviridae* family, order *Nidoviridales* [2].

Pigs mount a rapid antibody response to infection by PRRSV, but the antibodies are mainly directed to the N- and M-proteins and are non-neutralizing [3]. The primary neutralization epitope of some North American PRRSVs is located in the middle of the glycoprotein 5 (GP5) ectodomain [4]. A truncated GP5 without the signal peptide sequence or the predicted transmembrane regions (residues 60–130) is able to elicit protective antibodies capable of detecting PRRSV-infected cells and of distinguishing this virus from others [5]. Currently, killed-virus and modified-live PRRSV vaccines are used to control PRRS. However, both of these types of vaccines have inherent drawbacks and the development of novel PRRSV vaccines is urgently needed [6, 7].

Recent advances in innate immunity research have indicated that pathogen-associated molecular patterns (PAMPs) are promising molecular adjuvants for subunit vaccines [8, 9]. Flagellin, the structural component of the flagellar filament in various locomotive bacteria, is a ligand for toll-like receptor 5 (TLR-5) in host cells [10, 11]. An increasing number of studies have demonstrated the effectiveness of flagellin as an adjuvant [12, 13], and flagellin is an effective inducer of innate immune effectors, such as cytokines and nitric oxide, thereby stimulating the activation of adaptive immune responses [14].

In the present study, we cloned a truncated rGP5 gene and constructed the fusion protein rGP5-FljB using a prokaryotic system. We then determined the TLR-5-specific bioactivity of fusion protein rGP5-FljB by detecting the expression levels of the cytokine interleukin 8 (IL-8) in HEK293-mTLR5 cells by sandwich enzyme-linked immunosorbent assay (ELISA). Last, we assessed the immunogenicity of rGP5 and the adjuvant properties of FljB in a mouse immunization assay. ELISA-based detection of GP5-specific and PRRSV-specific antibodies suggested that FljB could enhance the immunogenicity of GP5 and induce a robust humoral immune response, thus providing more effective antibodies against PRRSV.

## Results

### Construction of expression plasmids bearing rGP5 and rGP5-fljB

**Fig. 1:**
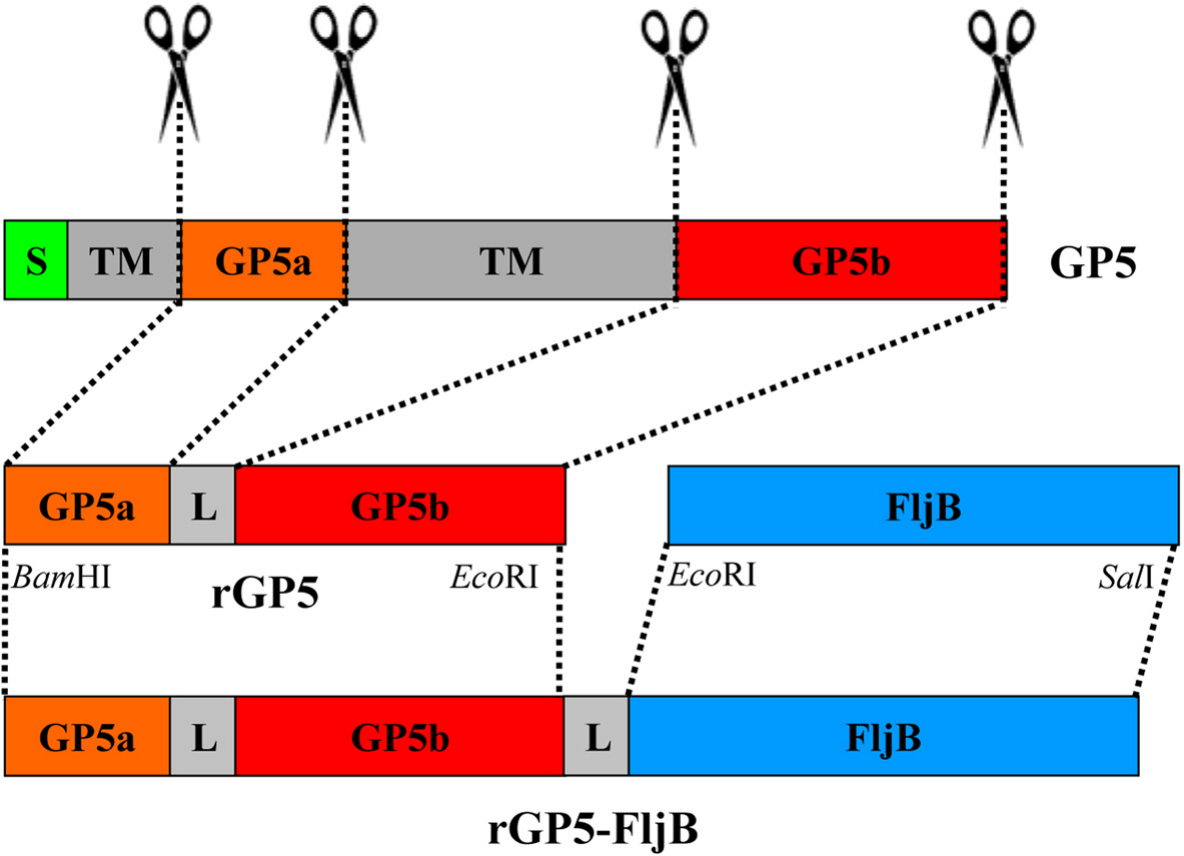
Schematic representation for the construction of rGP5 and rGP5-fljB. rGP5 fragment with the deletion of its signal peptide (green) and transmembrane regions (light grey) was amplified by overlap-PCR, and inserted into the *Bam*HI and *Eco*RI digested expression vector pColdI or pGEX-6p-1 to create pCold-rGP5 or pGEX-6p-1-rGP5 respectively. Flagellin *fljB* gene (light blue) was amplified from the genomic DNA of attenuated *Salmonella* Typhimurium SL7207 strain and cloned into the *Eco*RI and *Sal*I sites of pCold-rGP5, resulting in a recombinant plasmid pCold-rGP5-fljB. “L” represents the linker sequence GGGGS

A truncated rGP5 gene lacking the signal peptide sequence and transmembrane regions was amplified using a linker-based overlap-PCR strategy. The gene of interest was inserted into the expression plasmids pColdI and pGEX-6p-1 to add a His or GST tag, respectively. Recombinant plasmid pCold-rGP5-fljB was constructed by using enzymes to digest the plasmid pCold-rGP5 and inserting the PCR product *fljB* (Fig. 1). The DNA sequencing results indicated that the sequences of the inserts were identical to the template sequences.

### Expression and purification of rGP5 and rGP5-FljB proteins

**Fig. 2:**
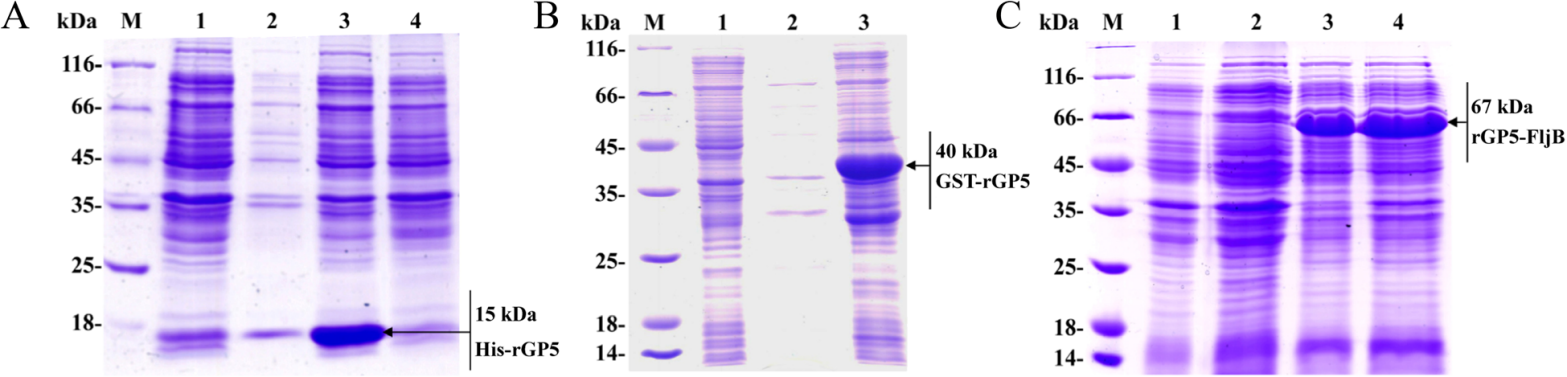
SDS-PAGE analysis of recombinant bacteria of BL21 (DE3)(pCold-rGP5) (**a**), BL21(DE3)(pGEX-6p-1-rGP5) (**b**) and BL21 (DE3)(pCold-rGP5-fljB) (**c**). (**a**) Lanes: M, molecular weight markers; 1, product of BL21(DE3)(pCold) induced by IPTG; 2, Lysate supernatant of bacteria bearing His-rGP5 induced by IPTG; 3, Inclusion bodies of bacteria bearing His-rGP5 induced by IPTG; 4, product of bacteria bearing His-rGP5 not induced. (**b**) Lanes: M, molecular weight markers; 1, product of BL21(DE3)(pGEX-6p-1) induced by IPTG; 2, Lysate supernatant of bacteria bearing GST-rGP5 induced by IPTG; 3, Inclusion bodies of bacteria bearing GST-rGP5 induced by IPTG. (**c**) Lanes: M, molecular weight markers; 1, product of BL21(DE3)(pCold) induced by IPTG; 2, product of bacteria bearing rGP5-FljB not induced; 3, Lysate supernatant of bacteria bearing rGP5-FljB induced by IPTG; 4, Inclusion bodies of bacteria bearing rGP5-FljB induced by IPTG

**Fig. 3:**
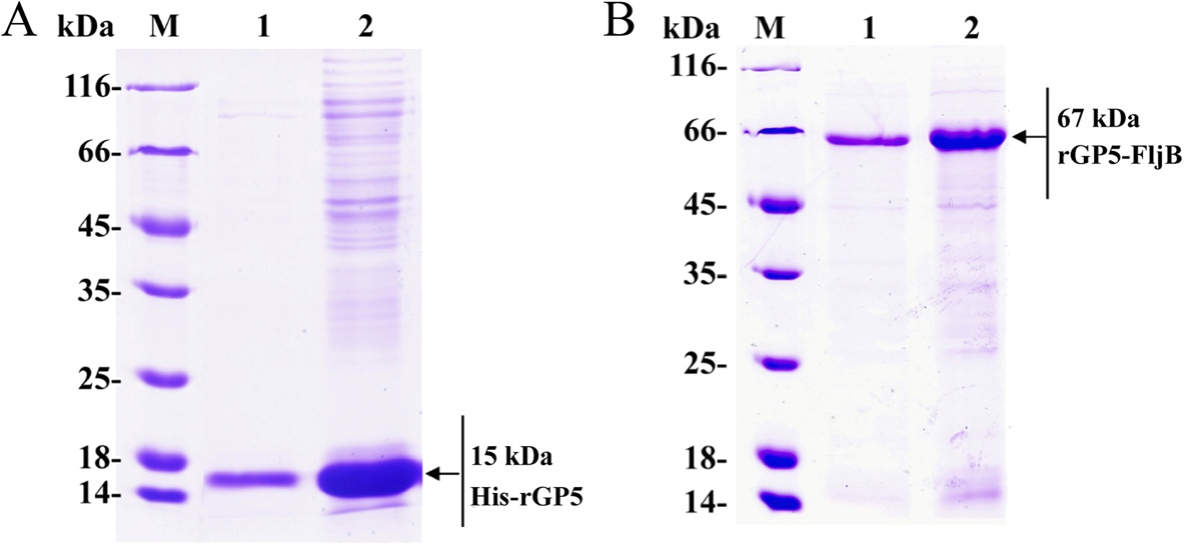
SDS-PAGE analysis of purified His-rGP5 and rGP5-FljB proteins. The fused His-rGP5 (**a**) and rGP5-FljB (**b**) were purified via Ni-NTA affinity columns. Lanes: M, molecular weight markers; 1 and 2, purified His-rGP5 or rGP5-FljB proteins

The expression of His-rGP5, GST-rGP5, and rGP5-FljB was induced by addition of IPTG to the culture medium. SDS-PAGE results indicated that the molecular weights of the tagged proteins were about 15 kDa for His-rGP5, 40 kDa for GST-rGP5, and 67 kDa for rGP5-FljB, as expected (Fig. 2). His-rGP5 and rGP5-FljB were purified via a Ni-NTA affinity column and verified by SDS-PAGE analysis (Fig. 3).

### Immunoblotting

**Fig. 4:**
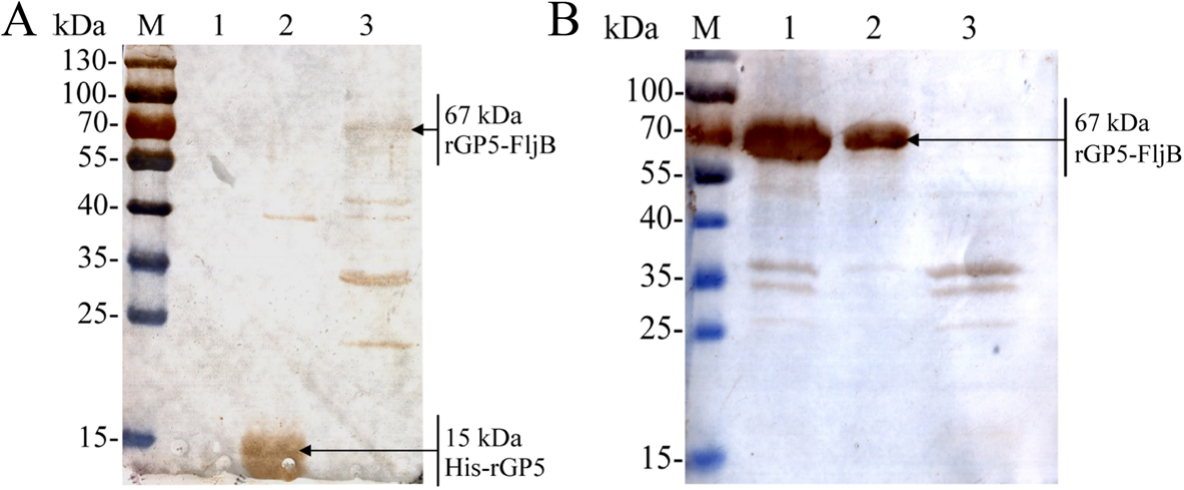
Western blotting analysis of His-rGP5 and rGP5-FljB proteins. (**a**) Analysis of His-rGP5 and rGP5-FljB with an anti-PRRSV polyclonal antibody. Lanes: M, molecular weight markers; 1, 1 × SDS-loading buffer; 2, purified His-rGP5 protein; 3, purified rGP5-FljB protein. (**b**) Analysis of rGP5-FljB with an anti-FljB polyclonal antibody. Lanes: M, molecular weight markers; 1, Lysate supernatant of BL21(DE3)(pCold-rGP5-FljB) induced by IPTG; 2, Inclusion bodies of BL21(DE3)(pCold-rGP5-FljB) induced by IPTG; 3, product of BL21(DE3)(pCold) induced by IPTG

To analyze the immunoreactivity of the rGP5 and fusion protein rGP5-FljB, polyclonal antibodies against PRRSV or FljB were used as the primary antibodies in a western blotting assay. The results showed that the bacterially-expressed rGP5 and rGP5-FljB were able to react with the PRRSV-specific antibody (Fig. 4a). Additionally, western blotting with anti-FljB antibodies confirmed the presence of a highly purified 67 kDa protein corresponding to rGP5-FljB (Fig. 4b).

### TLR-5 activation by purified fusion protein rGP5-FljB

**Fig. 5:**
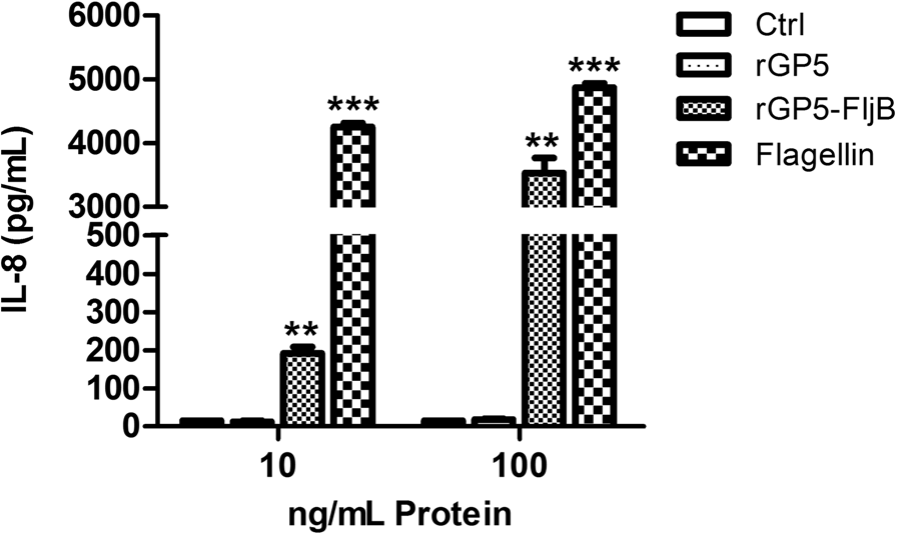
TLR5-specific activity of recombinant fusion proteins. The TLR5-specific activity of recombinant flagellin fusion protein was examined on the HEK293-mTLR5 cell line expressing mouse TLR5. Cells were treated with endotoxin-free recombinant proteins rGP5 or rGP5-FljB at the concentration of 10 and 100 ng/ml for 5 h. For positive controls, HEK293-mTLR5 cells were treated with the TLR5 agonist Flagellin. Supernatants were collected and expression levels of IL-8 were then evaluated by ELISA. Error bars indicate standard deviations of the means. Statistical significance was determined at *p* < 0.01 (**) or *p* < 0.001 (***)

After removal of endotoxin from the purified proteins rGP5 and rGP5-FljB, very low endotoxin levels, less than 0.03 EU/μg, were detected in the protein preparations, indicating that LPS contamination in the prepared proteins was negligible. To verify the activation of innate immune signaling by fusion protein rGP5-FljB, we conducted a TLR-5-specific bioactivity assay. Stimulation with rGP5-FljB at several different protein concentrations elicited a robust production of IL-8 that was significantly higher than that elicited by stimulation with similar concentrations of rGP5 alone (*p* < 0.01; Fig. 5). The observed up-regulation of chemokine IL-8 following stimulation with rGP5-FljB suggested that the prepared endotoxin-free rGP5-FljB was able to activate the innate immune response *in vitro*.

### Effects of rGP5-FljB treatment on GP5-specific and PRRSV-specific serum antibodies

**Fig. 6:**
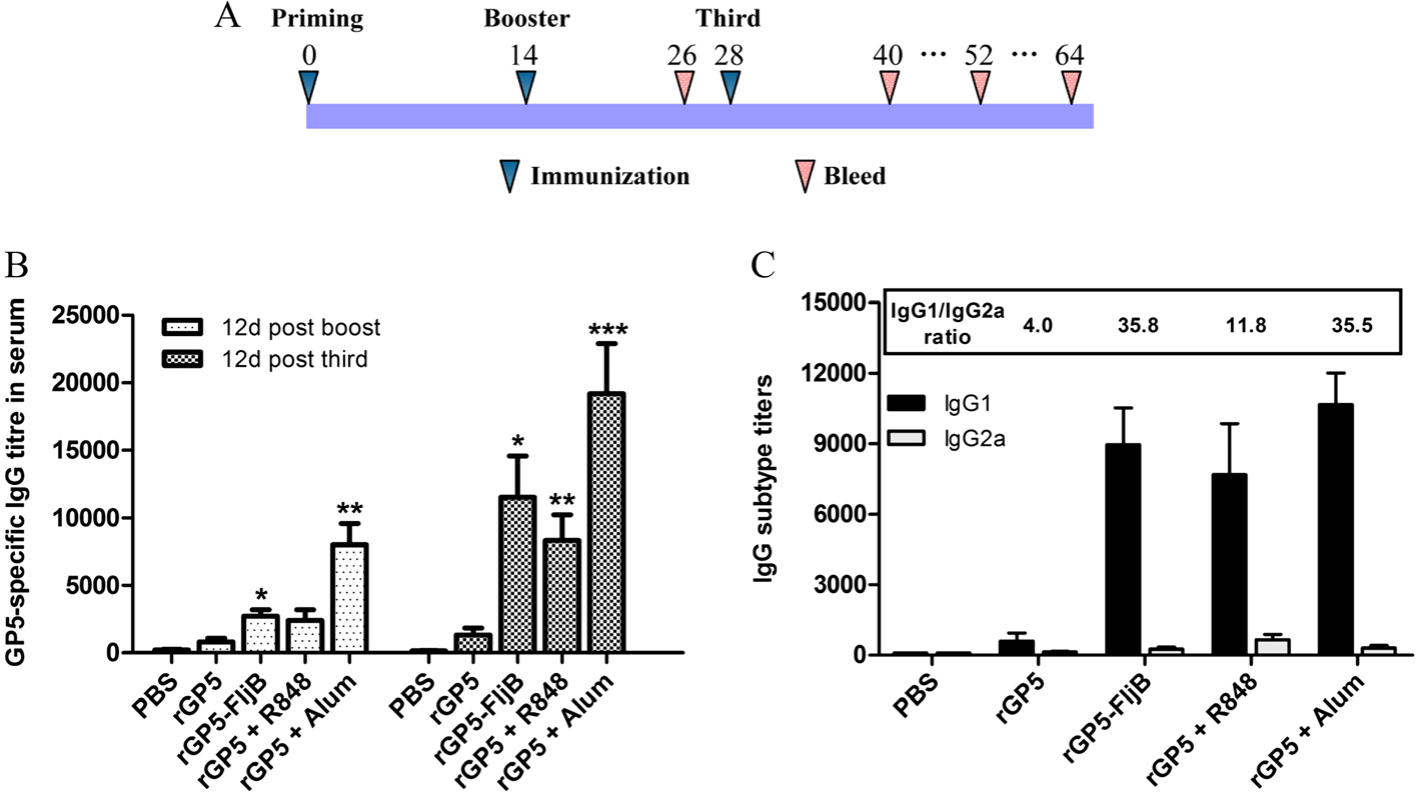
Immunization schedule and GP5-specific IgG antibody titers in serum. (**a**) C3H/HeJ mice were randomly divided into five groups (6 mice per group) and immunized intraperitoneally either with rGP5, rGP5-FljB, rGP5 + R848, rGP5 + aluminium adjuvant, or PBS, respectively. These mice were immunized three times on days 0, 14, and 28 at a dose of 50 μg rGP5, 50 μg rGP5-FljB, 10 μg R848 or isochoric aluminium adjuvant in 200 μL. Blood was collected from eye sockets on days 26, 40, 52 and 64 for analysis of anti-GP5 IgG titers by ELISA. (**b**) GP5-specific IgG antibody titers in serum at day 12 after the boost and third immunization. (**c**) IgG1 and IgG2a levels in serum at day 12 after the third immunization. Data reflects the mean ± SD by using the Student *t* test at *p* < 0.05 (*), *p* < 0.01 (**) or *p* < 0.001 (***)

**Fig. 7:**
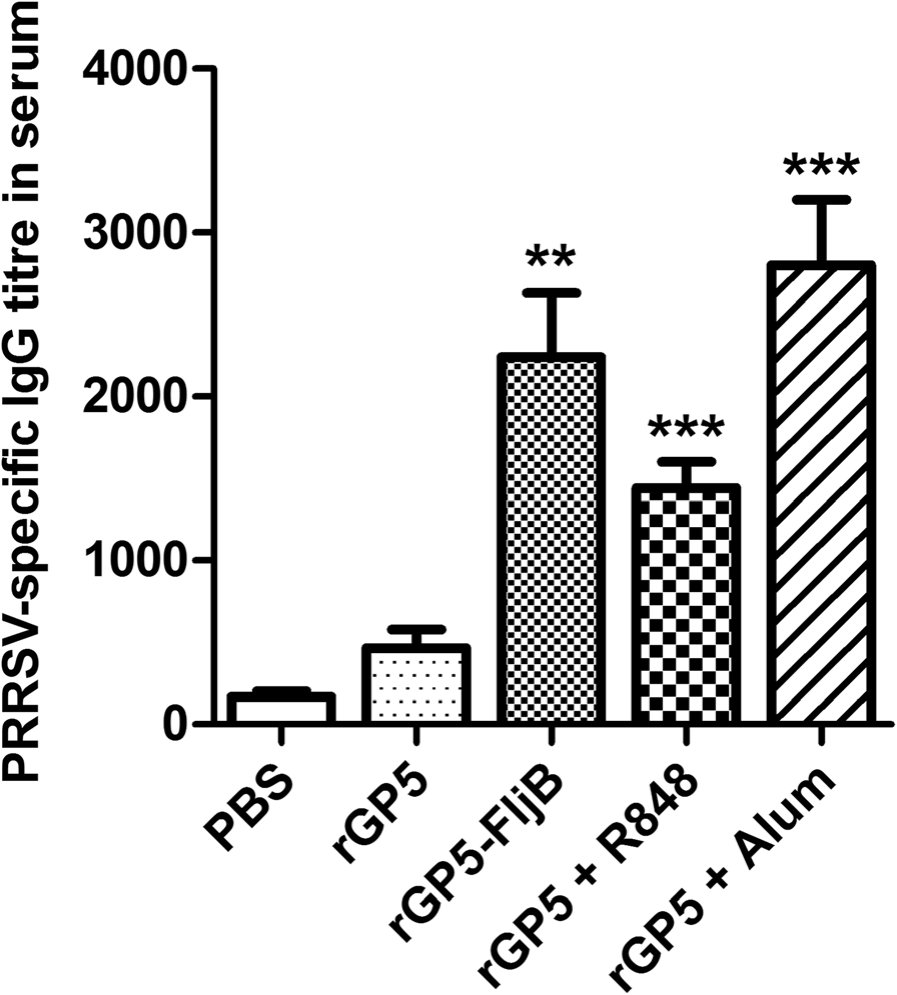
PRRSV-specific IgG antibody titers in serum at day 12 after the third immunization. C3H/HeJ mice were randomly divided into five groups (6 mice per group) and immunized intraperitoneally either with rGP5, rGP5-FljB, rGP5 + R848, rGP5 + aluminium adjuvant, or PBS, respectively. These mice were immunized three times on days 0, 14, and 28 at a dose of 50 μg rGP5, 50 μg rGP5-FljB, 10 μg R848 or isochoric aluminium adjuvant in 200 μL. Blood was collected from eye sockets on days 40 for analysis of anti-PRRSV IgG titers by ELISA. Data reflects the mean ± SD by using the Student *t* test at *p* < 0.01 (**) or *p* < 0.001 (***)

To examine the antibody response to the recombinant protein in an animal model, we administered the endotoxin-free fusion protein rGP5-FljB via an intraperitoneal injection. The results showed that immunization with the fusion protein induced a significantly enhanced GP5-specific and PRRSV-specific IgG response after three immunizations compared with immunization using rGP5 alone (Figs. 6b and 7). The titer in the rGP5-FljB immunization group was elevated almost four-fold after the third immunization compared with the titer after the second immunization. Co-administration of the rGP5 with R848 or Alum also yielded a higher IgG response than immunization with rGP5 alone (Fig. 6b).

### Immune subtype induced by fusion protein rGP5-FljB

We investigated the subtype of the immune response after three immunizations with fusion protein rGP5-FljB by detecting serum IgG1 and IgG2a via indirect ELISA. Co-administration of rGP5 with R848 or Alum induced a robust Th2 immune response. The IgG1/IgG2a ratio in the group of mice immunized with rGP5 alone was 4.0, whereas in the group of mice immunized with rGP5 together with R848 or Alum this ratio was 11.8 or 35.5, respectively. The IgG1/IgG2a ratio in the rGP5-FljB immunization group was significantly higher (9-fold) than that in the rGP5 alone immunization group, and reached a level equivalent to the IgG1/IgG2a ratio in the rGP5 + Alum immunization group (Fig. 6c), suggesting a strong Th2 immune response.

### The longevity of GP5-specific serum IgG

**Fig. 8:**
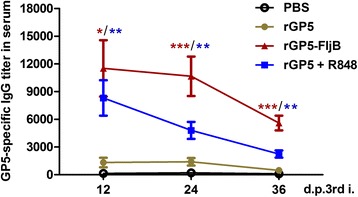
The longevity of GP5-specific serum IgG titers. Mice were bled for 36 days at 12 days intervals after 3rd immunization, and serum IgG levels were determined by indirect ELISA. The ELISA plates were coated with GST-taged GP5 antigen. The time course of GP5-specific antibodies induced by rGP5 alone, rGP5-FljB or rGP5 + R848 was analyzed. Data reflects the mean ± SD by using the Student *t* test at *p* < 0.05 (*), *p* < 0.01 (**) or *p* < 0.001 (***)

To monitor the longevity of the GP5-specific serum IgG, immunized animals were bled at 12-day intervals for 36 days after the third immunization and serum IgG levels were determined by indirect ELISA. The ELISA plates were coated with GST-tagged GP5 antigen. We analyzed the time course of the levels of GP5-specific antibodies induced by rGP5 alone, rGP5-FljB, or rGP5 + R848 (Fig. 8). The IgG titer in the rGP5-FljB group reached its peak (11,520) on day 12 post-third immunization, and then gradually fell. The rGP5-FljB immunization induced an approximately 7–12 fold increase in the GP5-specific IgG titers compared with the rGP5 alone immunization over the different tested time points, and the IgG titer of the rGP5-FljB group was still significantly higher than that of the rGP5 alone group on day 36 post-third immunization. These results suggest that rGP5-FljB immunization produces both a higher and longer-lasting anti-GP5 IgG titer than immunization with rGP5 alone.

## Discussion

PRRSV infection has been recognized as a severe threat to the pig industry. A hallmark of the swine antibody response against PRRSV is the abundant nonneutralizing antibodies detected early in infection followed by a low neutralizing antibody titer that appears at least 3 weeks after infection [15, 16]. The viral glycoprotein can induce a potent humoral immune response, and the production of anti-GP5 antibody is associated with the disappearance of viremia [4].

The *Escherichia coli* (*E. coli*)-based prokaryotic expression system is a powerful host cell system for expressing heterologous genes [17]. Many glycoproteins from cells or viruses produced in this system have shown biological activities [18, 19]. Therefore, we aimed to express a truncated rGP5 gene [5] without the signal peptide sequence and transmembrane regions in *E. coli*. The fused His-rGP5 was purified via a Ni-NTA affinity column by its His-tag (Fig. 3a). The immunoreactivity of rGP5 was determined by western blotting, and the results show that the heterologously-expressed protein reacts with an anti-PRRSV polyclonal antibody (Fig. 4a), indicating that the recombinant protein retained the biological activity of the wild-type protein.

TLRs recognize distinctive ligands, play key roles in innate immunity, and can contribute to the development of appropriate adaptive immune responses [20]. Recently, several TLR ligands have been widely studied as adjuvants for immunotherapy and vaccination [8, 21]. Among the defined TLR agonists, flagellin is the only protein with defined genetic codes [22]. To investigate the adjuvant activity of fusion protein rGP5-FljB, phase II flagellin *fljB* was amplified from the *S.* Typhimurium SL7207 strain and the recombinant plasmid pCold-rGP5-fljB was constructed. The fusion protein preparation had a high degree of purity, as only one protein band was observed (Fig. 3b). Flagellins from Gram-negative bacteria undergo a direct interaction with the leucine-rich regions in TLR-5 and activate a range of inflammatory cells via a TLR-5-dependent signaling pathway [11, 12]. We conducted a TLR-5-specific bioactivity assay and the results showed that stimulation with fusion protein rGP5-FljB induced a significantly higher expression of IL-8 than stimulation with rGP5 alone, at several different protein concentrations (Fig. 5), indicating that the innate immune response was activated by flagellin FljB.

Recently, an ever increasing number of studies have described the adjuvant property of flagellin in the context of a broad range of recombinant vaccines [23, 24]. We tested the ability of rGP5-FljB to act as an adjuvant by administering the fusion protein rGP5-FljB to mice, and found that immunization with the fusion protein induced a significantly-enhanced GP5-specific and PRRSV-specific IgG response compared with immunization with rGP5 alone (Figs. 6b and 7), indicating that the polyclonal antibodies against rGP5 were able to react with PRRSV and FljB is an efficacious adjuvant for the induction of antigen-specific IgG production.

Imiquimod, a synthetic TLR-7 agonist, can expedite the immune response against influenza virus infection when combined with influenza vaccines [25]. In this study, the GP5-specific IgG titer following rGP5 + R848 co-administration was significantly upregulated after the third immunization (Fig. 6b) and persisted for almost 5 weeks (Fig. 8). The robust IgG1/IgG2a ratio post-third immunization in the rGP5-FljB group, similar to that of the rGP5 + R848/Alum co-administration groups, indicated that a strong GP5-specific Th2 response was induced by the fusion protein rGP5-FljB (Fig. 6c).

The intranasal administration of other antigens in combination with flagellin has been shown to significantly increase the antigen-specific IgA titer, not only in the mucosal compartment but also in the serum [10]. Additionally, previous studies found that immunization via the intranasal but not the subcutaneous route elicited sporozoite neutralizing antibodies capable of inhibiting > 90 % of sporozoite invasion [26]. These findings suggest that a comparison of different administration routes of flagellin is needed in future studies.

## Conclusions

We designed a truncated rGP5 gene and prepared His-rGP5, GST-rGP5, and rGP5-FljB proteins with a prokaryotic expression system. The immunoreactivites of His-rGP5 and fusion protein rGP5-FljB were confirmed by western blotting. After the removal of endotoxin in our rGP5 and rGP5-FljB preparations, we verified the activation of an innate immune response via a TLR-5-specific bioactivity assay and investigated the adjuvant activity of FljB via administration of rGP5-FljB protein to mice. Our findings confirm that FljB could serve as an excellent immunoadjuvant for the production of GP5-specific and PRRSV-specific IgG antibodies and the induction of a robust humoral immune response.

## Methods

### cDNA amplification

Viral RNA was extracted from the Chinese isolate HH08 of PRRSV using TRIzol reagent (Invitrogen, CA, USA), and viral cDNA was synthesized using Oligo dT primers (Takara, Dalian, China) according to the manufacturer’s instructions. Based on the GP5 sequence of PRRSV (GenBank Accession number: GQ184821), primers were designed for amplifying a fragment covering the ORF5 gene of PRRSV (Table 1). PCR was carried out using PrimeSTAR HS DNA Polymerase (Takara) in a 50-μl reaction volume consisting of 1× PrimeSTAR Buffer (Mg^2+^ plus), 200 μM of each dNTP, 0.2 μM of forward and reverse primers, 1.25 U of PrimeSTAR HS DNA Polymerase, and ~200 ng of viral cDNA template. PCR amplifications were performed as follows: 1 cycle of 98 °C for 5 min, then 25 cycles of 98 °C for 10 s, 60 °C for 15 s, and 72 °C for 90 s, followed by 1 cycle of 72 °C for 10 min. The amplified PCR product was purified, cloned into pCR2.1-T with a TA Cloning Kit (Invitrogen) according to the manufacturer’s instructions, and sequenced by GenScript (Nanjing, China).

**Table 1:** PCR primers used in this study

Primer name	Primer sequence (5′ → 3′)	Application
GP5-ORF F	ACTTAAGCTTGGTACCATGG	Amplification of GP5 ORF
GP5-ORF R	CGCTAGAGCGCTGGCAAGTG	Amplification of GP5 ORF
GP5a F	CCGGGATCCAACGCCAGCGACAACAAC	Amplification of GP5a fragment
GP5a R	**CACCGCCGCTTCCACCGCCACC**CTCCACTGCCCAGTCAAA	Amplification of GP5a fragment
GP5b F	**GTGGAAGCGGCGGTGGCGGAAGC**TGCATGTCCTGGCGCTA	Amplification of GP5b fragment
GP5b R	CCGGAATTCGAGACGACCCCATAGTTCCGCT	Amplification of GP5b fragment
fljB F	GGGGGAATTC **GGTGGCGGTGGTTCT**ATGGCACAAGTAATCAACACTAACAGT	Amplification of fljB fragment
fljB R	GCGTCGACTTAACGTAACAGAGACAGC	Amplification of fljB fragment

### Construction of recombinant plasmids

The GP5 ectodomain with the deletion of its signal peptide and transmembrane regions was designated according to a previously published protocol with minor modifications [5]. The truncated rGP5 was amplified by overlap-PCR with two pairs of primers (Table 1). *Bam*HI and *Eco*RI restriction enzyme sites were introduced in GP5a-F and GP5b-R, respectively (underlined parts). A linker sequence encoding two repeated amino acid sequences (GGGGS) was introduced into primers GP5a-R and GP5b-F (linker sequence in black bold). GP5a and GP5b fragments were amplified by PCR using GP5a-F/GP5a-R and GP5b-F/GP5b-R, respectively, as described above except with an extension for 30 s at 72 °C. The resulting PCR products were electrophoresed on 1 % agarose gels and purified as the template of the truncated rGP5 gene amplification using primers GP5a-F and GP5b-R. The rGP5 fragment was inserted into the *Bam*HI and *Eco*RI-digested expression vector pColdI or pGEX-6p-1 to create pCold-rGP5 or pGEX-6p-1-rGP5, respectively. The phase II flagellin *fljB* gene was amplified from the genomic DNA of attenuated *Salmonella* Typhimurium SL7207 strain using primers fljB-F and fljB-R (Table 1). The purified PCR product was digested and cloned into the *Eco*RI and *Sal*I sites of pCold-rGP5, resulting in a recombinant plasmid pCold-rGP5-fljB (Fig. 1). The sequences of the resulting constructs were confirmed by DNA sequencing.

### Expression and purification of PRRSV rGP5 and rGP5-FljB

Recombinant plasmids pCold-rGP5, pGEX-6p-1-rGP5, and pCold-rGP5-fljB were purified with plasmid purification kits (Takara) according to the manufacturer’s instructions and transformed into host cells, *E. coli*, BL21(DE3) pLysS. Protein expression was optimized according to a recently published method with minor modifications [18]. Briefly, the *E. coli* harboring pCold-rGP5, pGEX-6p-1-rGP5, or pCold-rGP5-fljB were cultured in LB liquid medium at 37 °C with shaking until the optical density (OD) of the culture at 600 nm reached 0.6. Then, isopropyl-β-d-thiogalactoside (IPTG) was added to a final concentration of 0.5 mM to induce expression at 25 °C for 5 h (pGEX-6p-1-rGP5) or at 15 °C for 24 h (pCold-rGP5 and pCold-rGP5-fljB). The empty vector-transformed bacteria were used as a control. The bacteria were pelleted by centrifugation at 10,000 rpm, at 4 °C for 10 min and re-suspended in TE buffer (50 mM Tris and 1 mM EDTA, pH 8.0). Next, they were digested with lysozyme at a final concentration of 100 μg/L at room temperature for 30 min. The cell suspension was sonicated on ice for 30 min. Then, the lysate was centrifuged at 10,000 rpm for 10 min at 4 °C. The supernatant and the pellets were each mixed with sodium dodecyl sulfate (SDS)-loading buffer. Both samples were subjected to 12 % SDS-polyacrylamide gel electrophoresis (SDS-PAGE). The purification of rGP5-FljB was performed by using a His•Bind Purification Kit (Novagen, USA) according to the manufacturer’s instructions, and His-rGP5 inclusion bodies were conducted under the condition of 6 M urea. The purified proteins of interest were designated as His-rGP5 or rGP5-FljB. The protein contents were determined with a Bradford assay and by SDS-PAGE analyses.

### Western blotting

The antisera against PRRSV or FljB were generated by immunizing mice with the inactivated PRRSV strain HH08 or FljB protein mixed with Freund's adjuvant. The purified recombinant proteins of rGP5 and rGP5-FljB were both subjected to SDS-PAGE and then transferred to a nitrocellulose membrane. The membranes were blocked with blocking buffer (5 % non-fat dry milk and 0.05 % Tween-20 in phosphate-buffered saline, PBST) at 4 °C overnight. The next day, the membranes were incubated with a polyclonal antibody against PRRSV or FljB (1:1000 diluted in PBST) at 37 °C for 2 h. After washing three times with PBST, the membranes were incubated with a horseradish peroxidase (HRP)-conjugated secondary antibody (1:5000 diluted in PBST, Boster, China) at 37 °C for 1 h. The protein bands were visualized via a diaminobenzidine enzyme-based color development in the dark that was terminated by distilled water.

### Endotoxin removal of rGP5 and rGP5-FljB

Contaminating lipopolysaccharide (LPS) was removed from the recombinant proteins His-rGP5 and rGP5-FljB by using the ProteoSpin™ Endotoxin Removal Kit Maxi for protein & peptides (Norgen, Canada) according to the manufacturer’s instructions, and the residual LPS content of the protein was measured using a chromogenic end-point tachypleus amebocyte lysate (CE TAL) assay kit (Chinese Horseshoe Crab Reagent Manufactory Co., Ltd., Xiamen, China) according to the manufacturer’s instructions.

### TLR-5-specific bioactivity assay

The detection of IL-8 expression levels in a HEK293-mTLR5 cell line (a gift from Dr. Yi Li, Baylor College of Medicine, Houston, USA) was used to test for the TLR-5-specific activity of the fusion protein rGP5-FljB. Briefly, HEK293-mTLR5 cells were cultured in 96-well microtiter plates (Corning, USA) at a seeding density of 5 × 10^4^ cells in 100 μl/well of DMEM medium supplemented with 10 % fetal calf serum and antibiotics. The next day, cells were treated with endotoxin-free recombinant proteins rGP5 or rGP5-FljB at concentrations of 10 and 100 ng/ml for 5 h. For positive controls, HEK293-mTLR5 cells were treated with the TLR-5 agonist flagellin (Sigma-Aldrich, USA). Supernatants were collected and expression levels of IL-8 were then evaluated using Human IL-8 Ready-SET-Go!® ELISA Set (eBioscience, San Diego, CA, USA).

### Mouse immunization

Six-week-old female C3H/HeJ mice were purchased from the Biomedical Research Institute of Nanjing University. They were housed in isolators and fed a pathogen-free diet and water. The procedures described in this study were approved by the Committee on the Ethics of Animal Experiments of Yangzhou University, Yangzhou, China. To test whether vaccination with the fusion protein rGP5-FljB provides a greater immune response than vaccination with rGP5 alone or with other adjuvants, C3H/HeJ mice were randomly divided into five groups (6 mice per group) and immunized intraperitoneally either with rGP5, rGP5-FljB, rGP5 + R848 (Imidazoquinoline; Enzo Life Sciences, USA), rGP5 + aluminum adjuvant (Alum, Thermo, USA), or PBS. These mice were immunized three times on days 0, 14, and 28 at doses of 50 μg rGP5, 50 μg rGP5-FljB, 10 μg R848 or isochoric aluminum adjuvant in 200 μL. Blood was collected from eye sockets on days 26, 40, 52, and 64 (Fig. 6a) and the sera were stored at—70 °C until they were tested by ELISA to determine their antibody levels.

### Detection of anti-GP5 and anti-PRRSV antibodies

Serum IgG, IgG1, and IgG2a titers against GP5 or IgG titers against PRRSV were measured by ELISA according to a previously described protocol with minor modifications [27]. Briefly, 96-well microtiter plates were coated with recombinant GST-GP5 (0.2 μg/ml) or purified inactivated PRRSV strain HH08 (0.5 μg/ml) in 50 mM carbonate buffer (pH 9.6) at 4 °C overnight and blocked for 2 h at 37 °C with blocking buffer (1 % bovine serum albumin in PBST). After washing three times with PBST, sera were added at an initial dilution of 1:100 with a two-fold dilution series in blocking buffer and incubated for 2 h at 37 °C. After five washes with PBST, antigen-specific antibodies were detected using goat anti-mouse IgG conjugated to HRP (1:10,000 dilution) or goat anti-mouse IgG1 and IgG2a conjugated to HRP (1:3000 dilution) for 1 h at 37 °C. The ELISAs were developed using 3,3≠,5,5 ≠ -Tetramethylbenzidine and H_2_O_2_ as substrates, and the ODs were read at 450 nm (A450) with an ELISA reader (Bio-Tek EL 680, USA).

### Statistical analysis

The significance of the difference between groups of fused or mixed proteins and rGP5 alone was determined by a Student’s *t*-test with Instat version 5.0 (GraphPad Software, San Diego, CA). Statistical significance was determined at *p* < 0.05 (*), *p* < 0.01 (**) or *p* < 0.001 (***).

## Abbreviations

PRRS: Porcine reproductive and respiratory syndrome
GP5: Glycoprotein 5
PAMPs: Pathogen-associated molecular patterns
TLR-5: Toll-like receptor 5
His: Histidine
IPTG: Isopropyl-β-d-thiogalactoside
SDS-PAGE: Sodium dodecyl sulfate-polyacrylamide gel electrophoresis
PBS: Phosphate-buffered saline
HRP: Horseradish peroxidase
LPS: Lipopolysaccharide
ELISA: Enzyme-linked immunosorbent assay
